# Social distancing policies in 22 African countries during the COVID-19 pandemic: a desk review

**DOI:** 10.11604/pamj.supp.2020.37.1.27026

**Published:** 2020-12-14

**Authors:** Andre Verani, Catherine Clodfelter, Akshara Narayan Menon, Jennifer Chevinsky, Kerton Victory, Avi Hakim

**Affiliations:** 1 U.S. Centers for Disease Control and Prevention, Atlanta, USA

**Keywords:** Severe acute respiratory syndrome coronavirus 2, COVID-19, Africa South of the Sahara, public health, public policy, pandemics

## Abstract

**Introduction:**

on January 30, 2020, the World Health Organization declared the novel coronavirus outbreak a Public Health Emergency of International Concern. As of October 5, 2020, there were over 34.8 million reported cases of severe acute respiratory syndrome coronavirus 2 (SARS-CoV-2) infection and more than 1 million reported deaths from coronavirus disease 2019 (COVID-19), globally. Non-pharmaceutical interventions, such as social distancing policies, hand hygiene, and mask use, are key public health measures to control COVID-19. In response to, or in some cases even before, the first wave of SARS-CoV-2 infections were reported in their countries, policy makers across Africa issued various social distancing policies.

**Methods:**

we describe social distancing policies issued from March 1 to April 24, 2020 in 22 Anglophone countries of sub-Saharan Africa. We reviewed policies identified online.

**Results:**

though all 22 countries closed schools and banned gatherings, they took a variety of approaches to sizes of gatherings banned and to stay-at-home orders, with 13 countries issuing national stay-at-home orders, four issuing subnational stay-at-home orders, and five not issuing stay-at-home orders. Enforcement provisions varied by country, as did funeral and health care exceptions.

**Conclusion:**

movement restrictions, business restrictions, and school closures can have substantial negative impacts on economies, education, nutrition, and routine health care. Yet easing or lifting of COVID-19 social distancing policies can lead to increased transmission. Our review documents a wide variety of policy alternatives used in Africa and can inform future adjustments as countries ease, lift, and reapply measures in response to their evolving epidemics.

## Introduction

On January 30, 2020, the WHO declared the novel coronavirus outbreak a Public Health Emergency of International Concern [1]. As of October 5, 2020, there were over 34.8 million reported cases of SARS-CoV-2 infection and more than 1 million reported deaths from COVID-19 disease globally [2]. By October 2020, vaccines were under development but not yet available. In response to the COVID-19 pandemic, governments around the world instituted social distancing policies such as closing schools, closing businesses, restricting gatherings, and recommending or ordering people to stay home [3]. Non-pharmaceutical interventions (NPI) include but are not limited to social distancing policies. As the name suggests, NPI are interventions other than pharmaceuticals (e.g. drugs or vaccines). During the response to COVID-19, NPI have included, in addition to social distancing policies, testing, contact tracing, quarantine of the exposed, isolation of the sick, infection prevention and control, personal protective equipment for health workers, and use of face coverings by members of the public. NPI including school closures and gathering bans reduced mortality during the influenza pandemics of 1918-19 and 2009-10 especially when interventions were instituted early, were layered (i.e. multiple simultaneous NPI) and were sustained [4-6]. NPI are also impacting the current COVID-19 pandemic. Early studies on measures adopted in response to COVID-19 have analyzed the impact of NPI on mobility and cases. A study in the United States found that mandated NPI were followed by community mobility reductions [7]. Lower community mobility may result in reduced opportunity for exposure, which may in turn lead to reduced SARS-CoV-2 transmission [8]. A more recent study estimated that COVID-19 cases would have been 35 times greater in the United States from March 1 to April 27, 2020, if state and local governments had not adopted the four social distancing measures studied: large event bans, school closures, business closures, and stay-at-home orders [9]. Another modeling study analyzing the relative effect of restrictions of inter-city population movement, early identification and isolation of cases, and social distancing measures found that the combined effect of multiple NPI was to reduce COVID-19 cases by 98.5% (1/67 of prior) in China up to February 29, 2020, compared to a scenario without NPI [10]. However, mandated NPI and additional measures such as travel and trade restrictions to control COVID-19 can negatively impact education, income, and other aspects of health [11]. Information about NPI during the early stages of COVID-19 in Africa is scarce. We provide detailed policy information about social distancing policies in 22 Anglophone countries in sub-Saharan Africa during the initial wave of COVID-19.

## Methods

We reviewed binding policies requiring social distancing from 22 English-speaking countries in the WHO Africa region: Botswana, Eritrea, Eswatini, Ethiopia, Gambia, Ghana, Kenya, Lesotho, Liberia, Malawi, Mauritius, Namibia, Nigeria, Rwanda, Seychelles, Sierra Leone, South Africa, South Sudan, Tanzania, Uganda, Zambia, and Zimbabwe [12]. We selected these countries based on the availability of policies in English, our primary language. We considered a policy to be binding if it was government issued and appeared to require, prohibit, or authorize actions. Social distancing has been defined by the CDC as, “keeping a safe space between yourself and other people who are not from your household ... at least 6 feet ...” [13]. We focused our study on policies requiring social distancing by either prohibiting individuals from leaving their homes or by prohibiting or restricting close contact with others outside their homes (e.g. at schools, at businesses, at events). Confronted with the challenge of researching policies in 22 distinct countries, and no single database that compiled texts of COVID-19 related policies across these countries, we developed a methodology that sought to balance uniformity across all countries and flexibility to allow for variations by country.

First, we developed a database to organize and describe policies by country and type of provision, including emergency declarations, gathering bans, school closures, business closures, stay-at-home orders (i.e. lockdowns), nighttime curfews, effective date, duration, exceptions, and enforcement. Second, we cross-referenced global and regional compilations of COVID-19 measures to ensure comprehensiveness of policies retrieved, including: Assessment Capacities Project (ACAPS) [14], GitHub [15], Africa Centres for Disease Control and Prevention (Africa CDC) [16], International Center for Not-for-profit- Law (ICNL) [17], and the U.S. Centers for Disease Control and Prevention (CDC) International Task Force´s global mitigation database.Third, we employed search terms based on measures identified in the compilations (e.g. “Kenya curfew”) and used the Google search engine to identify policy texts publicly available online at official government databases and government postings on social media, supplemented by credible non-governmental sources. Policies found included legislation, rules, regulations, orders, and other documents with language suggesting their binding nature (e.g. “shall”). We abstracted relevant provisions into the database. Fourth, we conducted quality checks with a second researcher reviewing the first researcher´s abstractions, reading the applicable policies, and the two reconciling any discrepancies. Our analysis covers the period between March 1 and April 24, 2020 (Table 1) as no COVID-19-related social distancing policies were issued prior to this period, to the best of our knowledge. By April 24^th^, all 22 countries had instituted multiple, social distancing policies. Thus, we considered that end date as capturing the first phase or iteration of such policies. Finally, we analyzed changes in community mobility by country from March 1 to April 30, 2020 in relation to these policies. These data were from anonymous users of Google location services, aggregated, and made publicly available. According to Google, these “community mobility reports aim to provide insights into what has changed in response to policies aimed at combating COVID-19. The reports chart movement trends over time by geography, across different categories of places such as retail and recreation, groceries and pharmacies, parks, transit stations, workplaces, and residential” [18]. Community Mobility Reports for the period we assessed were available for 12 of 22 countries. For each country, we calculated median change in mobility from the baseline of January 3 to February 6 to the study period of March 1 to April 30, using Microsoft Excel (2007). The baseline is from before we might expect to see changes in community mobility due to COVID-19 related NPIs, and the study period is when we might expect to see changes in community mobility due to COVID-19 related NPIs.

**Table 1:** COVID-19 social distancing policies timeline in 22 African countries

Country	First case confirmed	Emergency declaration	Schools closed	Gatherings restricted	Businesses restricted	Stay-at-home order
Botswana	April 1	March 20	April 2	March 16	April 2	April 2
Eritrea	March 22	NA	March 27	March 23	March 23	April 2
Eswatini	March 15	March 17	March 17	March 17	March 27	March 27
Ethiopia	March 14	April 9	March 16	March 16	April 11	NA
Gambia	March 19	March 27	March 18	March 17	March 27	NA
Ghana	March 14	March 21	March 15	March 15	March 23	March 30*
Kenya	March 14	NA	March 15	March 22	March 22	NA
Lesotho	May 14	March 27	April 4	April 4	April 4	April 4
Liberia	March 17	March 21	March 21	March 21	March 21	April 10
Malawi	April 3	March 20	March 20	March 20	March 20	NA
Mauritius	March 19	March 19	March 19	March 23	March 23	March 23
Namibia	March 15	March 17	March 16	March 14	March 14	March 28
Nigeria	February 28	NA	March 26	March 30	March 30	March 30*
Rwanda	March 16	NA	March 16	March 14	March 21	March 21
Seychelles	March 16	March 20	April 8	March 30	March 30	April 8
Sierra Leone	April 1	March 24	March 31	March 16	April 5	April 5
South Africa	March 6	March 15	March 15	March 15	March 26	March 26
South Sudan	April 6	NA	March 20	March 20	March 28	NA
Tanzania	March 17	NA	March 17	March 17	NA	NA
Uganda	March 22	NA	March 20	March 16	March 16	March 30
Zambia	March 19	NA	March 20	March 14	March 14	April 15*
Zimbabwe	March 21	March 23	March 30	March 23	March 30	March 30

* Subnational stay-at-home orders such as Nigeria's at state and Zambia's at district levels

Confirmed first cases from https://who.int/emergencies/diseases/novel-coronvirus-2019/situation-reports

## Results

All 22 countries mandated multiple social distancing measures in order to control COVID-19. Annex 1 lists social distancing policies reviewed by country, with links to publicly available online sources. Nineteen of the 22 countries responded to COVID-19 with one or more social distancing policies either before (9 countries) or within one week (10 countries) of their first confirmed case (Table 1). Emergency or disaster declarations and declarations of “infected areas” often authorized subsequent restrictions (e.g. on movement of persons). For example, South Africa´s disaster declaration authorized issuance of subsequent regulations including the nationwide stay-at-home order. We found emergency or disaster declarations for 14 countries (Botswana, Eswatini, Ethiopia, Gambia, Ghana, Lesotho, Liberia, Malawi, Mauritius, Namibia, Seychelles, Sierra Leone, South Africa, Zimbabwe). Additionally, at least 4 countries had provisions authorizing declarations of infected areas (Kenya, Liberia, Uganda, Zambia). Gathering bans and school closures were implemented across the 22 countries, and at least 21 countries closed non-essential businesses (Tanzania may have closed non-essential businesses in one or more policies written in the Kiswahili language). Seventeen countries had a national and/or subnational lockdown (i.e. stay-at-home order), and five had not issued a stay-at-home order although two of these did have a nighttime curfew (Table 2). Gathering bans and school closures were often instituted before stay-at-home orders (Table 1). An example of the stepwise policy-making process comes from Liberia where the Minister of Health declared a National Health Emergency and designated two counties as infected areas with gathering restrictions, school, and business closures implemented in just those two counties. Just over two weeks later, the President declared a State of Emergency for the entire country, which was followed by a stay-at-home order first applicable to five counties but later extended to the entire country (Table 1).

**Table 2:** summary of COVID-19 social distancing policies in 22 African countries

Measure	Countries
School closures	22 (Botswana, Eritrea, Eswatini, Ethiopia, Gambia, Ghana, Kenya, Lesotho, Liberia, Malawi, Mauritius, Namibia, Nigeria, Rwanda, Seychelles, Sierra Leone, South Africa, South Sudan, Uganda, Tanzania, Zambia, Zimbabwe)
Business closures	21 at least (Tanzania policy not found in English language but may be in Kiswahili language)
Gathering bans	22
National lockdowns (i.e. stay-at-home orders)	13 (Botswana, Eritrea, Eswatini, Lesotho, Liberia from April 24, Mauritius, Namibia, Rwanda, Seychelles, Sierra Leone, South Africa, Uganda, Zimbabwe)
Subnational lockdowns (i.e. stay-at-home orders)	4 (Ghana, Liberia from April 10, Nigeria, Zambia)
No lockdowns (i.e. no stay-at-home orders)	6 (Ethiopia, Gambia, *Kenya, **Malawi, *South Sudan, Tanzania)

* Nighttime curfews; **National lockdown prohibited by judiciary. See The High Court of Malawi, Kathumba & Ors. v. The President & Ors. (Judicial Review Cause No. 22 of 2020) MWHC 8, April 28, 2020, re. March 17 injunction

Business restrictions were more difficult to categorize since each country closed or otherwise regulated businesses in a unique manner. Contrasting Zambia and Zimbabwe is instructive in this regard. In Zambia, restaurants were limited to take-away and delivery service, bars, night clubs, cinemas, gyms, and casinos were closed, and specifically in Kafue District (declared an “infected area”) all shops were closed one day for disinfection by health officials. Contrast this to Zimbabwe, where every business was closed except for those providing essential services, and essential services were listed in the policy. Also excepted were restaurants attached to hotels and those providing food for off-premises consumption. Finally, Zimbabwe required COVID-19 diagnostic testing of employers and employees in non-essential businesses prior to returning to work for the first time during the phased relaxation of the national lockdown. Other noteworthy business restrictions included closure of weekly markets in Eritrea, designation of businesses as low or high-risk and corresponding restrictions in Eswatini, prohibition of liquor sales in Lesotho, closure of barbershops and beauty salons in Liberia, demarcation of 1.5 meters of distance between persons inside and outside of shops in Namibia, restriction of business hours in Seychelles, and limitation of retail sales to food, water, medicine, fuel and other essential commodities in Sierra Leone.

**Figure 1:**
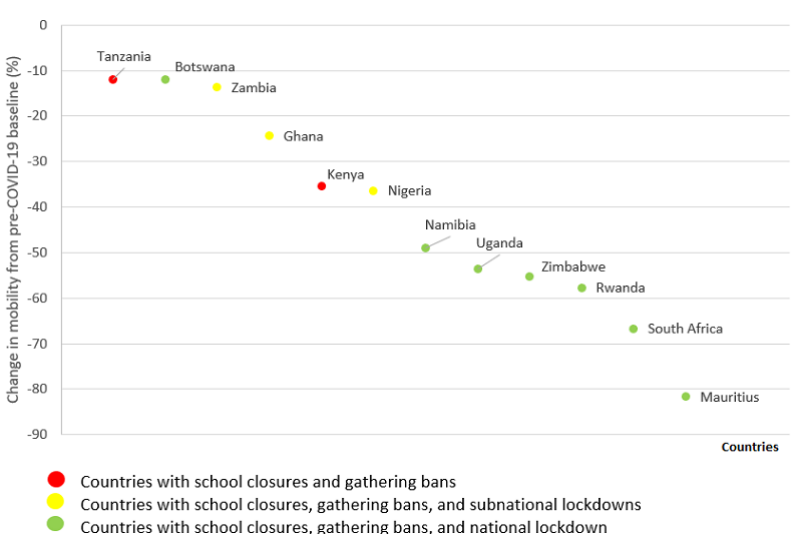
median percent change in community mobility from pre-COVID-19 baseline (January 3 - February 6, 2020) to study period (March 1 - April 30, 2020) in 12 of 22 African countries (google location data)

Stay-at-home orders had exceptions for essential goods and/or services including health care (Table 3). Health care provision and access exceptions to stay-at-home orders varied by country, with some countries stating how persons are to comply with the policy and other countries not doing so. Broadly speaking, we found three groups of approaches. In the first group (Eritrea, Eswatini, Ghana, Liberia, Nigeria, Rwanda, Seychelles, Uganda, Zambia, and Zimbabwe), stay-at-home policies provided no guidance for how health workers and patients were to prove they are moving outside their homes in order to provide or access health care. In the second group of countries (Botswana, Mauritius, Namibia, and Sierra Leone), stay-at-home policies required permits for health workers and patients to move freely outside their homes. In the third group of countries (Lesotho, Malawi, and South Africa), stay-at-home policies required permits for health workers but provided no guidance for patients. Enforcement provisions for the policies reviewed often included fines or prison. Of the 16 countries with stay-at-home orders, 14 countries´ policies called for punishing non-compliance with fines or prison and/or specifically noted which entities were responsible for enforcement (Table 4). Some countries also regulated COVID-19 related information with enforcement provisions, such as Lesotho prohibiting the publication or spread of fake or false information, punishable by fine and/or prison. In addition to capturing the first phase or iteration of social distancing policies beginning in March, we found that by April, some countries such as Eswatini and Sierra Leone had started easing or lifting stay-at-home orders. This was indicative of the beginning of a global trend [16]. As far as changes in community mobility data are concerned, we found that median community mobility decreased from baseline during March 1-April 30, 2020 in all 12 countries for which data were available, with a wide range of median decreases by country from 12% in Tanzania [range 3% to -36%] to 82% in Mauritius [range 10% to-92%] (Figure 1). These decreases reflect changes in personal behavior (i.e. reduced mobility of individuals) some of which may be attributable to policy mandates. Decreases in median community mobility were greater in countries with national lockdowns (except Botswana) than in countries without national lockdowns.

**Table 3:** COVID-19 social distancing policy variations in 22 African countries

Issue	Country	Illustrative provisions
Health Care Access Exception to stay-at-home orders allowing for movement outside of one's home to provide or to access essential goods and/or services	Botswana	Form A required for people to access essential services (e.g. patients) and Form B for people providing essential services (e.g. health workers).
	Lesotho	Letter of designation required for persons performing essential services.
	Nigeria	Documentation or proof for patients or health workers is not explicitly addressed in the law.
	Zimbabwe	Every individual found outside his or her home shall have the burden of proof.
Gathering Ban Thresholds	Eswatini	Gatherings restricted to 20 persons (e.g. religious activities, sports events, conferences, wedding celebrations, music concerts, parties, gymnasiums, or other activities or place where the public gathers).
	Ethiopia	Gatherings restricted to 4 persons who do not belong to a single family, in any place for religious, government, social or political purposes (but sports and games in public are banned with no numerical threshold).
	Ghana	Suspends all public gatherings including funerals and religious activities including in churches and mosques.
	Sierra Leone	Public gatherings restricted to 100 persons.
Funeral Attendance Exception to Gathering Ban Thresholds	Ghana	Private burials with up to 25 attendees allowed.
	Kenya	Funerals restricted to immediate family members.
	Rwanda	Funerals restricted to 10 persons. Places of worship closed.
	South Africa	Funerals restricted to 50 persons. Permit can be issued for family members to travel to funeral.
Business Closures	South Sudan	Closure of businesses selling non-essential commodities, ban on hawking, and limitation of workday to half day.
	Uganda	Closure of bars, night-clubs, gyms, saunas, public swimming pools and hair-salons but no closure of stores selling general merchandise except in shopping malls.
	Zambia	Restaurants only take-away and delivery. Closed bars, night clubs, cinemas, gyms, and casinos.
	Zimbabwe	Every business closed except essential services (listed)

**Table 4:** enforcement provisions in 16 African countries with stay-at-home orders

Country	Enforcement provisions
Botswana	fines, up to 6 months prison
Eritrea	police, security, neighborhood committees
Eswatini	security forces, chiefs, traditional authorities, community police
Ghana	patrols, snap checks, roadblocks by police, military, other security services
Lesotho	fines, up to a month prison, defense force and mounted police
Liberia	armed forces and security forces
Mauritius	fines, up to 6 months prison
Namibia	fines, up to 6 months prison
Nigeria	fines, up to 6 months prison
Rwanda	local government institutions and security organs
Seychelles	officer may direct person to residence, remove person to residence, or arrest
Sierra Leone	none explicit
South Africa	fines, up to 6 months prison
Uganda	up to 3 months prison
Zambia	none explicit
Zimbabwe	fines, up to a year prison

## Discussion

Our review describes COVID-19 social distancing policies across multiple African countries. Since policies are likely to change over the course of the pandemic, it can help provide an initial baseline for study. This knowledge is relevant for at least two reasons. First, it documents the widespread use of multiple social distancing policies by African leaders to help structure their governments´ responses to COVID-19. Second, our paper details various examples of the range of policy provisions across these 22 countries, including areas of uniformity (e.g. existence of gathering bans) and areas of divergence (e.g. size of gatherings banned ranging from 2-100 persons), both of which can inform future policy choices. A common pattern emerged with many countries first banning gatherings and closing schools before later issuing stay-at-home orders. It could be the case that policymakers across many of these countries thought that gathering restrictions and school closures, along with business restrictions, might be sufficient to control the worst effects of the pandemic without resorting to more restrictive stay-at-home orders with their potentially negative socio-economic impacts. However, most countries (73%) did eventually issue stay-at-home orders whether sub-nationally or nationwide.

School closures were the most uniform social distancing policy, with all countries closing schools from pre-primary to professional educational levels for both public and private schools even though policies were worded somewhat differently from country to country. A few examples follow. Eritrea´s policy stated, “All institutions of learning - from Kindergarten to Colleges - will be closed starting tomorrow, 27^th^ March 2020.” The Gambia´s policy stated, “All schools... will be closed from Wednesday, 18^th^ March 2020 for 21 days.” Ghana´s policy stated, “All Universities, Senior High Schools, and basic schools, i.e. public and private schools, will be closed Monday, 16^th^ March, 2020, till further notice.” (Table 1) Policy variations may have differential effects on COVID-19 mitigation efforts, as may varying approaches to enforcement. It is unclear to what extent the policies we reviewed have been implemented and enforced, and such a study is beyond our scope. However, enforcement issues including excessive use of force have been reported [19,20]. Examples of policy variation include numeric thresholds for gathering bans, funeral exceptions, and health care access exceptions allowing for patients and health workers to leave home during lockdowns (Table 3). The size of permitted gatherings diverged widely across time within countries and across countries. Countries commonly made their gathering bans stricter over time, as in Botswana where on March 16^th^ gatherings of over 100 persons were banned and four days later gatherings of 10 or more persons were banned. These varied widely across countries; Ghana suspended all public gatherings of 2 or more persons while Malawi and Sierra Leone allowed public gatherings of up to 100 people.

We found funerals and similar ceremonies for the deceased to be common exceptions to gathering bans and stay-at-home orders. The competing demands of controlling COVD-19 superspreading events such as funerals [21] and allowing for grieving and continuity of cultural ceremonies [22] is reflected in these countries´ policies. The number of permitted funeral attendees varied widely among the countries, such as Kenya restricting funeral attendees to immediate family members, Rwanda limiting funerals to 10 attendees, and South Africa allowing for up to 50 persons. Whereas Ghana banned all gatherings of 2 or more persons, an exception was made for private burials with up to 25 participants (Table 3). Gatherings during funerals in Sierra Leone were restricted to 20 family members. Variations in health care access exceptions to lockdown movement restrictions were related to how providers and patients were to prove to officials (if stopped in transit) that they were outside their homes in order to access health care. Some policies explicitly required permits or passes while other policies remained silent on this point (Table 3). Implementation of health care access exceptions may affect movement of persons providing and seeking health care during lockdowns, with potential ramifications for HIV patients, children in need of routine immunizations, and others.

Limitations: limitations of our study include exclusive reliance on online sources for policies researched and lack of in-country legal research and validation by lawyers authorized to practice in these 22 countries, as well as the cross-sectional nature of our policy research in a dynamic policy environment. This study does not measure the effects of policies, though we recognize the need for impact studies. Future research should assess epidemiological impacts of NPI on SARS-CoV-2 transmission and COVID-19 cases throughout Africa.

## Conclusion

The decision to issue an NPI through a binding policy is complex and difficult. On the one hand, movement restrictions, business restrictions, and school closures can have substantial negative impacts on economies [23], education [24], food access [25], and routine health care [26]. On the other hand, easing or lifting of COVID-19 social distancing policies can lead to increased transmission, as in second waves [27]. Studies cited earlier in our paper provide evidence that NPI such as school closures, gathering bans, business restrictions and stay-at-home orders have helped control COVID-19 by reducing community mobility. As national and subnational governments reapply, ease, and lift social distancing policies in response to their evolving epidemics, our review documents a wide variety of policy alternatives used in Africa. Understanding the range and variety of social distancing policies establishes a baseline, may help identify gaps and opportunities, and can inform future mitigation strategies in the Africa region.

**Disclaimer:** the findings and conclusions of this report are those of the authors and do not necessarily represent the official position of the U.S. Centers for Disease Control and Prevention.

### What is known about this topic

- Social distancing policies have reduced incidence and mortality in respiratory pandemics;
- Social distancing policies have reduced incidence of COVID-19, in countries studied;
- Social distancing policies have been applied throughout the world, in response to COVID-19.

### What this study adds

- 16 of 22 anglophone African countries instituted stay-at-home orders (i.e. lockdowns);
- 22 of 22 anglophone African countries closed schools and restricted gatherings;
- For 12 of 22 countries with data, decreases in median community mobility were greater in countries with national lockdowns (except Botswana) than in countries without this measure.

## Annex

**Annex 1**: list of and links to COVID-19 social distancing policies in 22 African Countries (PDF - 413Kb)
